# Complete plastome assemblies from a panel of 13 diverse potato taxa

**DOI:** 10.1371/journal.pone.0240124

**Published:** 2020-10-08

**Authors:** Sai Reddy Achakkagari, Maria Kyriakidou, Helen H. Tai, Noelle L. Anglin, David Ellis, Martina V. Strömvik

**Affiliations:** 1 Department of Plant Science, McGill University, Montreal, Canada; 2 Fredericton Research and Development Centre, Agriculture and Agri-Food Canada, Fredericton, Canada; 3 International Potato Center, Lima, Peru; National Cheng Kung University, TAIWAN

## Abstract

The chloroplasts are a crucial part of photosynthesizing plant cells and are extensively utilized in phylogenetic studies mainly due to their maternal inheritance. Characterization and analysis of complete plastome sequences is necessary to understand their diversity and evolutionary relationships. Here, a panel of thirteen plastomes from various potato taxa are presented. Though they are highly similar with respect to gene order and content, there is also a great extent of SNPs and InDels between them, with one of the *Solanum bukasovii* plastomes (BUK2) having the highest number of SNPs and InDels. Five different potato plastome types (C, S, A, W, W2) are present in the panel. Interestingly, the *S*. *tuberosum* subsp. *tuberosum* (TBR) accession has a W-type plastome, which is not commonly found in this species. The S-type plastome has a conserved 48 bp deletion not found in other types, which is responsible for the divergence of the S-type from the C-type plastome. Finally, a phylogenetic analysis shows that these plastomes cluster according to their types. Congruence between the nuclear genome and the plastome phylogeny of these accessions was seen, however with considerable differences, supporting the hypothesis of introgression and hybridization between potato species.

## Introduction

The chloroplasts are an essential part of all photosynthesizing plant cells. Chloroplast DNA, the plastome, is maternally inherited and has a higher degree of conservation than nuclear or mitochondrial DNA, in terms of gene order and content [1, 2]. The plastome is composed of a single circular DNA molecule, typically ranging between 115 and 165 kb in land plants [3]. Potato (*Solanum* sp.) plastomes range from 154 to 156 kb, with a typical quadripartite structure including one large single copy (LSC) region, one small single copy (SSC) region, and two copies of inverted repeat regions (IRa and IRb). The inverted repeats are ~25 kb in length and separated by the LSC (~86 kb) and SSC (~18 kb) regions [3, 4]. Despite the degree of plastome conservation in most land plants, many structural and single nucleotide variations have been observed. These variations include interspecies deletions [3], insertions and deletions (InDels), single nucleotide polymorphisms (SNPs), genome expansions, and polymorphic single sequence repeats (SSRs) [4, 5].

The taxonomy of wild and cultivated *Solanum* species is very complex and is continuously debated. The *Solanum* genus includes ornamentals and nightshades as well as food crops such as tomato, potato, and eggplant. A recent taxonomy study on potato—members of the tuber-forming clade of *Solanum—*reported that there are 107 wild and 4 cultivated potato species [6], instead of the 232 species previously proposed [7]. The plastome was used extensively in previous studies as phylogenetic markers [8], however, the plastome phylogeny does not necessarily follow the nuclear genome phylogeny [9]. Nonetheless, generating both phylogenies is useful in terms of studying cross-hybridization and introgression in potato [8].

Five basic plastome types (A, S, C, T, and W) were previously described in potato based on a restriction fragment length polymorphism (RFLP) analysis [10]. The W-type is considered the most primitive [10], since its restriction fragment pattern is closest to those of Mexican diploids as well as non-tuber-bearing *Solanum* species. The majority of the plastome types are derived from the W-type through various point mutations with the exception of the T-type, which is derived by a single deletion of 241 bp from the W-type [11]. The additional W1, W2, and W3 types were derived from the W-type by a single nucleotide change shown in the *PvuII* restriction fragment pattern, whereas the C-type was derived by a single change that led to a difference in the *BamHI* restriction fragment pattern [10, 11]. The A and S-type originated from the C-type through single nucleotide changes, evidenced through changes in the *BamHI* and *HindIII* restriction fragment patterns, respectively [10]. A diverse set of plastomes has been found among potato species as a result of successive domestication, polyploidization and selection [11, 12].

Just like mitochondrial DNA, plastid DNA can be used to study maternal genetic lineage. However, the use of partial plastome sequences was shown to be insufficient to classify closely related species [13], and it is therefore necessary to characterize complete plastomes to deduce phylogenetic relationships and evolution of species.

In this study, the plastomes from a panel of 13 potato accessions were characterized. The nuclear genomes of 12 of these accessions were previously studied for structural variation [14, 15], and in the present study we present their complete plastome sequences and from an additional individual wild potato accession. Furthermore, SNPs, InDels, repeat sequences present as well as the type of each plastome are identified based on polymorphisms. The topology obtained from a phylogenetic analysis of the complete plastomes of 16 accessions/species (13 from the panel and three reference sequences) is compared with the previously published topology obtained using the nuclear genomes.

## Materials and methods

### Plant materials, DNA library preparation, and sequencing

DNA was extracted from plants of twelve Peruvian potato accessions: *Solanum stenotomum* subsp. *goniocalyx* (GON1—CIP 702472), *S*. *stenotomum* subsp. *goniocalyx* (GON2—CIP 704393), *S*. *phureja* (PHU—CIP 703654), *S*. *xajanhuiri* (AJH—CIP 703810), *S*. *stenotomum* subsp. *stenotomum* (STN—CIP 705834), *S*. *bukasovii* (BUK1—CIP 761748), *S*. *bukasovii* (BUK2 –CIP 761748), *S*. *tuberosum* subsp. *andigena* (ADG1—CIP 700921), *S*. *tuberosum* subsp. *andigena* (ADG2—CIP 702853), *S*. *curtilobum* (CUR—CIP 702937), *S*. *juzepczukii* (JUZ—CIP 706050), *S*. *chaucha* (CHA- CIP 707129) and one Chilean accession: *S*. *tuberosum* subsp. *tuberosum* (TBR—CIP 705053) and sequenced using Illumina PE Technology as described in [14]. The genome of a second individual from the *S*. *bukasovii* (BUK2 –CIP 761748) accession, BUK2, was sequenced with 10X Genomics’ GemCode technology (https://www.10xgenomics.com/).

### *de novo* assembly and annotation

Raw reads obtained from Illumina sequencing were initially processed using Trimmomatic v0.36 to remove adapter sequences and low-quality bases [16]. TruSeq3 paired end Illumina adapters, low-quality bases, and reads less than 60 bp in length were removed. The parameters used for the Trimmomatic were ILLUMINACLIP: TruSeq3-PE. fa: 2:30:10 LEADING:3 TRAILING:3 SLIDINGWINDOW: 4:15 MINLEN:60. The reads of BUK2 obtained from the 10X Genomics’ GemCode technology were run through LongRanger *Basic* to perform read trimming and barcode error correction (https://support.10xgenomics.com/genome-exome/software/pipelines/latest/installation). The filtered reads of each landrace were assembled into a complete plastome using NOVOPlasty: *de novo* organellar genome assembler [17], which uses a seed-and-extend algorithm to *de novo* construct a complete plastome from whole genome sequencing (WGS) data. A seed sequence of 1000 bp was randomly selected from the doubled monoploid *S*. *tuberosum* Group *Phureja* clone DM1-3 plastome [18]. The expected genome range was set to 145 kb-165 kb, and other parameters set to default. Inverted repeat regions were detected in assembled sequences using REPuter software [19]. The assembled sequences were annotated using GeSeq [20] with *S*. *commersonii* (GenBank accession NC_028069.2) [4] and *S*. *tuberosum* (GenBank accession DQ231562.1) [3] species as references. The annotations were manually curated to adjust start and stop codons using Blastn searches [21]. The circular maps of all the plastomes were drawn using OGDRAW v1.3.1 [22]. The inverted repeats and gene structure in junction sites of all the plastomes were visualized using IRscope [23].

### Variant detection

SNPs and InDels were determined from the multiple sequence alignment of the thirteen plastomes. Multiple sequence alignments (MSA) were performed using MAFFT v7 with 1PAM/k = 2 scoring matrix [24]. SNPs were detected using SNP-sites [25]. InDels were detected using a custom python script and Geneious Prime 2019 (Geneious Prime 2019.2 https://www.geneious.com). From the MSA, a consensus sequence was obtained and annotated to determine the genes harboring the variant sites. Furthermore, at each variant site any base with a frequency of more than 50% was considered a reference allele. Gapped annotations were obtained from Geneious Prime 2019, and the variants were annotated using gapped annotations with BEDTools v2.28 [26].

### Repeat identification

Simple sequence repeats (SSRs) were identified using PHOBOS v3.3.12 [27] with a minimum number of repeat units as follows: 10 for mononucleotide repeats, 5 for dinucleotide repeats, 4 for trinucleotide repeats, and 3 for tetranucleotide repeats, pentanucleotide repeats, and hexanucleotide repeats. Tandem repeats were detected using Tandem Repeat Finder v4.09 [28]. Forward, palindromic, reverse, and complemented repeats present in all the plastomes were identified using REPuter software with minimum repeat size of 30 bp and a hamming distance of 3 [19].

### Identification of chloroplast DNA types

Two methods were used to identify plastome types. Method 1: Seven microsatellite markers and their PCR product sizes were used to determine the types [12, 29]. Marker analysis was conducted using primer sequences of these seven markers with an *in silico* PCR “reaction” [30]. Method 2: *In silico* restriction endonuclease analysis: the digestion sites for five restriction enzymes *Bam*HI, *Hind*III, *Kpn*I, *Pvu*II, and *Xho*I were used for an *in silico* restriction digest reaction. The digestion was performed *in silico* using RestrictionMapper (http://www.restrictionmapper.org/). Custom scripts were used to parse the output and compare the results amongst 13 plastomes to determine the length polymorphisms. These restriction fragment patterns were compared with the previous studies to determine the types [1, 10].

### Phylogenetic analyses

To understand the phylogenetic position of these plastomes, a maximum parsimony phylogeny was constructed using PAUP* version 4.0a167 with 1000 bootstrap replicates [31]. Published plastomes of *S*. *tuberosum* group Phureja DM1-3 516 R44 (DM) [18], *S*. *commersonii* (GenBank accession NC_028069.2) [4], and *S*. *tuberosum* (GenBank accession DQ231562.1) [3] were also included in the phylogeny. One inverted repeat region from each plastome was removed before constructing the phylogeny to avoid data duplication.

## Results and discussion

### Structure and organization of potato plastomes

**Fig 1:**
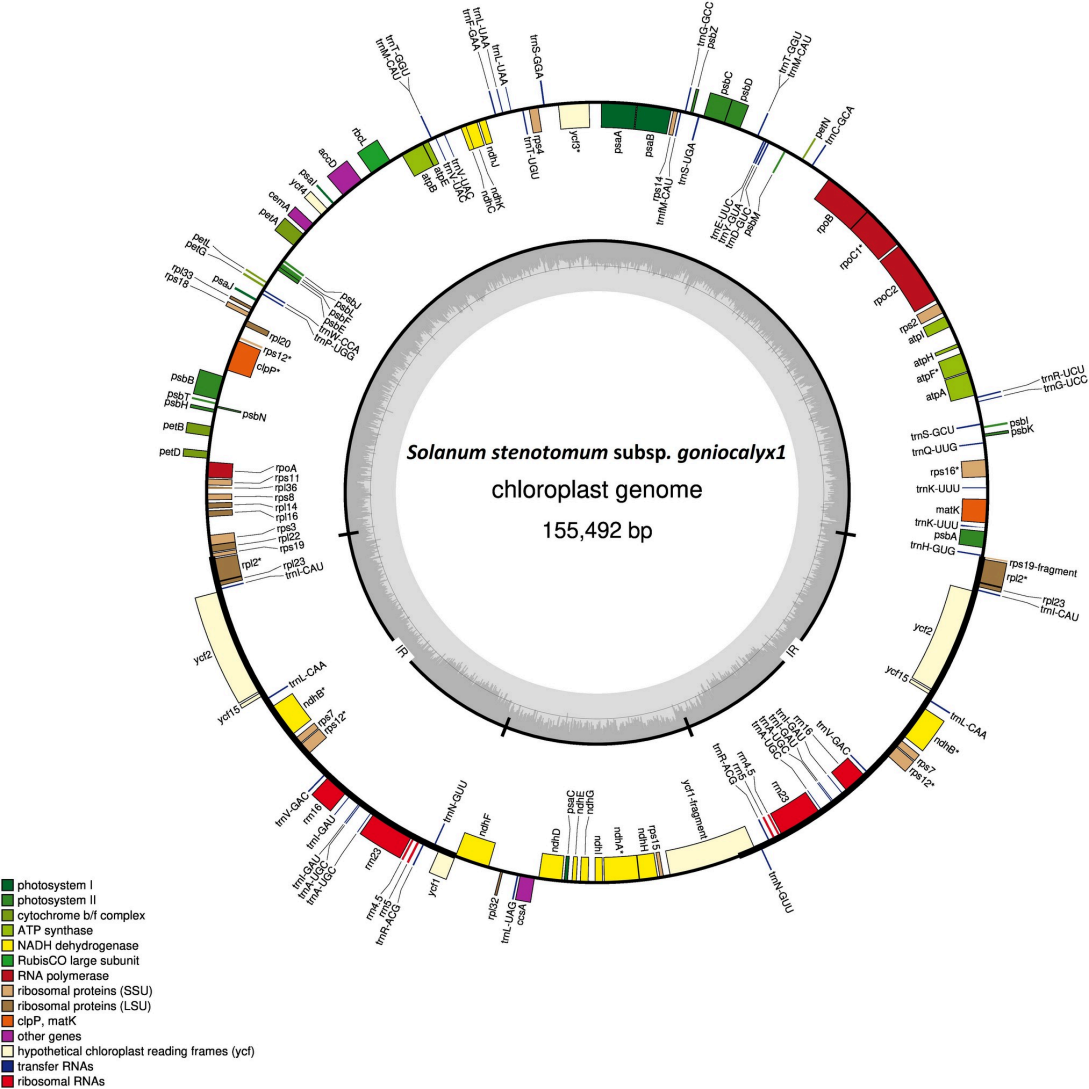
Gene structure of the *Solanum stenotomum* subsp. *goniocalyx* 1 plastome. Structure and gene content of the GON1 plastome. A panel of 13 potato plastomes all have similar gene content and structure. Genes that lie outside the circle are transcribed clockwise, genes inside the circle are transcribed counter-clockwise. The inner circle represents GC content.

Whole genomic DNA was previously sequenced from a panel of 12 potato accessions (Table 1) [14]. In this study, another *S*. *bukasovii* genome (BUK2) from the same accession as BUK1 was added to the panel, and their combined 13 plastomes were assembled and investigated to assess the diversity among them. For each potato accession, the entire plastome was assembled into a single circular sequence using trimmed WGS reads, with a typical quadripartite structure of a plastid DNA molecule: one large single copy (LSC), one small single copy (SSC), and two inverted repeat regions (IRa and IRb) (Fig 1). The sequencing coverage of each plastome ranges from 9,348 to 25,856 X. The size of the plastomes ranges between 155,486 bp (AJH) and 155,584 bp (BUK2) (Table 2), and they are all highly identical in structure and genome organization. Inverted repeat (IR) regions of 25,593 bp are found in 12 of the plastomes, while the plastome of BUK2 differs with 25,596 bp of IR regions (3 bp more per IR). The size of the LSC and SSC regions ranges from 85,929 bp to 86,005 bp and 18,363 bp to 18,394 bp respectively. The GON1, GON2, PHU, STN, and CUR plastomes have the same size and boundaries. Similarly, the CHA and ADG2 plastomes have the same size (Table 2). The GC content in the AJH and ADG1 plastomes is 37.8%, while it is 37.9% in the rest of them.

**Table 1:** A panel of 13 potato plastomes.

Potato Accession	Label	Nuclear ploidy level	CIP Accession identifier	Sequence coverage (X) on plastome	GenBank accession #
*Solanum stenotomum* subsp. *goniocalyx*	GON1	Diploid	702472	14,576	MT120855
*Solanum stenotomum* subsp. *goniocalyx*	GON2	Diploid	704393	25,856	MT120856
*Solanum xajanhuiri*	AJH	Diploid	703810	15,388	MT120857
*Solanum phureja*	PHU	Diploid	703654	15,995	MT120858
*Solanum stenotomum* subsp. *stenotomum*	STN	Diploid	705834	12,028	MT120859
*Solanum bukasovii*	BUK1	Diploid	761748	25,459	MT120860
*Solanum bukasovii*	BUK2	Diploid	761748	9,348	MT120867
*Solanum juzepczukii*	JUZ	Triploid	706050	14,319	MT120863
*Solanum chaucha*	CHA	Triploid	707129	11,628	MT120864
*Solanum tuberosum* subsp. *andigena*	ADG1	Tetraploid	700921	10,177	MT120861
*Solanum tuberosum* subsp. *andigena*	ADG2	Tetraploid	702853	12,789	MT120862
*Solanum tuberosum* subsp. *tuberosum*	TBR	Tetraploid	705053	9,873	MT120865
*Solanum curtilobum*	CUR	Pentaploid	702937	9,815	MT120866

The plastomes from a panel of 13 potato accessions with various nuclear ploidy levels including seven diploid, two triploid, three tetraploid, and one pentaploid were assembled. Two individuals, BUK1 and BUK2, from the same accession of the wild *S*. *bukasovii* were sequenced.

**Table 2:** Plastome size and range of individual segments.

Potato accession	Size (bp)	LSC (size bp)	IRb (size bp)	SSC (size bp)	IRa (size bp)
GON1	155,492	1–85,930 (85,930)	85,931–111,523 (25,593)	111,524–129,899 (18,376)	129,900–155,492 (25,593)
GON2	155,492	1–85,930 (85,930)	85,931–111,523 (25,593)	111,524–129,899 (18,376)	129,900–155,492 (25,593)
AJH	155,486	1–85,937 (85,937)	85,938–111,530 (25,593)	111,531–129,893 (18,363)	129,894–155,486 (25,593)
PHU	155,492	1–85,930 (85,930)	85,931–111,523 (25,593)	111,524–129,899 (18,376)	129,900–155,492 (25,593)
STN	155,492	1–85,930 (85,930)	85,931–111,523 (25,593)	111,524–129,899 (18,376)	129,900–155,492 (25,593)
BUK1	155,491	1–85,929 (85,929)	85,930–111,522 (25,593)	111,523–129,898 (18,376)	129,899–155,491 (25,593)
BUK2	155,584	1–85,998 (85,998)	85,999–111,594 (25,596)	111,595–129,988 (18,394)	129,989–155,584 (25,596)
JUZ	155,532	1–85,981 (85,981)	85,982–111,574 (25,593)	111,575–129,939 (18,365)	129,940–155,532 (25,593)
CHA	155,518	1–85,968 (85,968)	85,969–111,561 (25,593)	111,562–129,925 (18,364)	129,926–155,518 (25,593)
ADG1	155,530	1–85,971 (85,971)	85,972–111,564 (25,593)	111,565–129,937 (18,373)	129,938–155,530 (25,593)
ADG2	155,518	1–85,968 (85,968)	85,969–111,561 (25,593)	111,562–129,925 (18,364)	129,926–155,518 (25,593)
TBR	155,564	1–86,005 (86,005)	86,006–111,598 (25,593)	111,599–129,971 (18,373)	129,972–155,564 (25,593)
CUR	155,492	1–85,930 (85,930)	85,931–111,523 (25,593)	111,524–129,899 (18,376)	129,900–155,492 (25,593)

The plastomes were assembled into a complete circular molecule with four segments included, i.e. one large single copy region (LSC), one small single copy region (SSC), and two inverted repeats (IRa and IRb). The overall size ranges from 155,486 to 155,584 bp. GON1, GON2, PHU, STN, and CUR have the same size and boundaries, and ADG2 and CHA have the same. The other six plastomes vary in size and boundaries.

**Fig 2:**
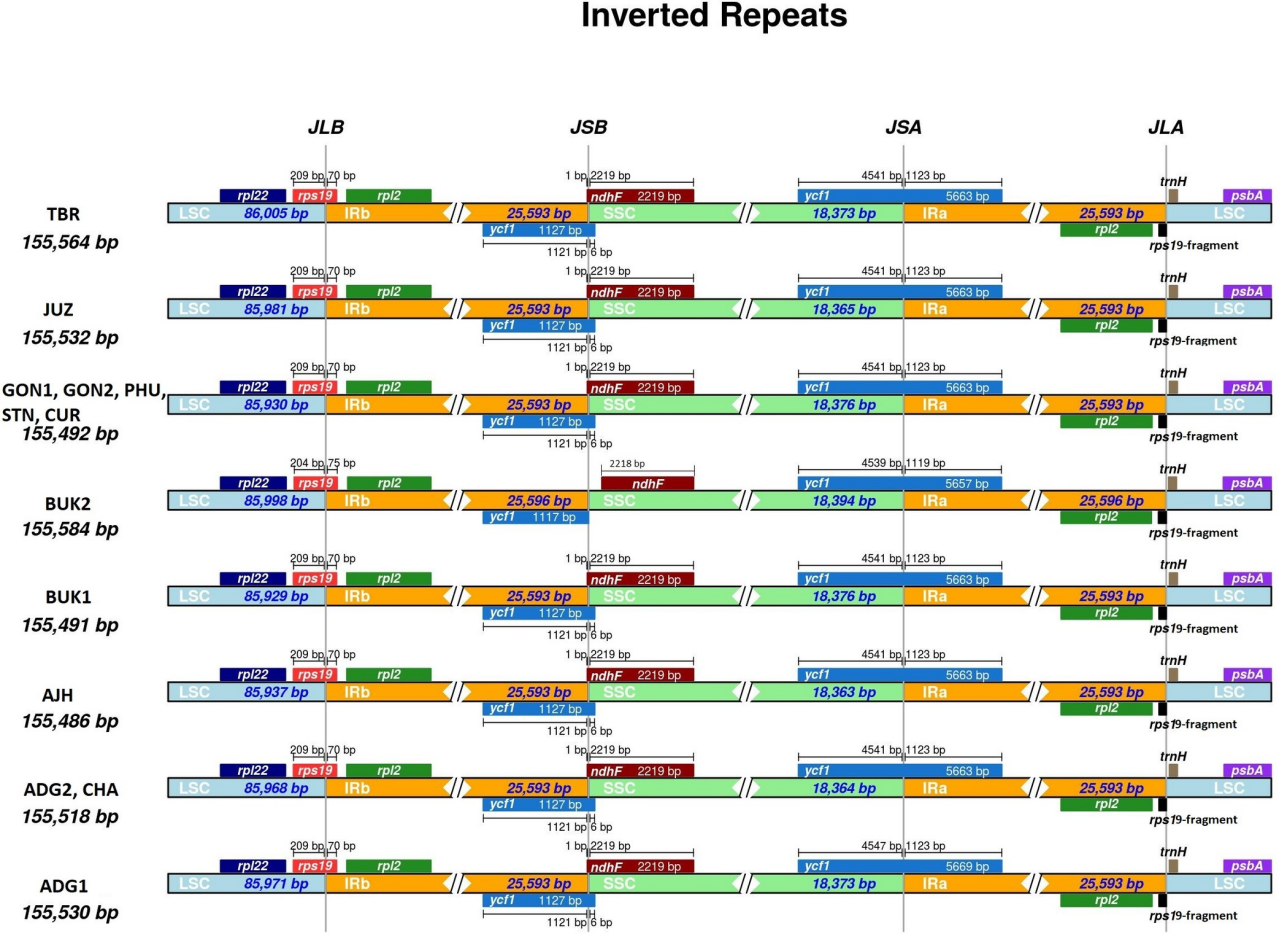
Representation of junction-sites in 13 potato plastomes. Four junction sites are present in all the plastomes, JLB (LSC-IRb junction), JSB (IRb-SSC junction), JSA (SSC-IRa junction), JLA (IRa-LSC junction). The BUK2 plastome differs from the other by the locations of the junctions.

Each plastome has 143 genes in total, of which 20 are present in both of the IR regions and five are duplicated in the LSC region (Table 3). Overall, 115 unique (single copy) genes are present in all the plastomes, of which 81 are protein coding genes, 30 are tRNAs, and four are the rRNAs. A total of 14 intronic regions are present in nine genes, of which five are found in the LSC region, three in IR regions, and one in the SSC region. The circular map of the GON1 plastome with the gene structure is shown in Fig 1. The gene structure at the inverted repeat junction sites is similar in all plastomes assembled here, with the exception of BUK2. The JLA (LSC-IRa junction site) is located upstream of the *rps19-fragment* and downstream of the *trnH-GUG* gene. The JLB (junction between LSC-IRb) is situated within the *rps19* gene. The junction between SSC and IRb is located within the *ycf1* and *ndhF* genes in all of them except in BUK2, where it is located in the intergenic region between these two genes. Furthermore, the JSA (SSC-IRa junction) is located within the *ycf1* gene (Fig 2). These 13 plastomes share similar gene structure, organization and gene boundaries with the previously sequenced *S*. *commersonii*, and *S*. *tuberosum* plastomes [3, 4]. However, the 13 plastomes each have more genes than those of *S*. *commersoni* and *S*. *tuberosum*, likely due to the usage of different annotation tools and curation methods. Nonetheless, the core set of genes and the unique protein coding genes, tRNAs, and rRNAs are the same in all of them.

**Table 3:** Distribution of genes in the plastomes.

Genome	Total Genes	Genes in LSC	IRb	SSC	IRa	Genes in junction sites	tRNAs	rRNAs	Introns
GON1	143	87	20	11	21	4	30	4	14
GON2	143	87	20	11	21	4	30	4	14
AJH	143	87	20	11	21	4	30	4	14
PHU	143	87	20	11	21	4	30	4	14
STN	143	87	20	11	21	4	30	4	14
BUK1	143	87	20	11	21	4	30	4	14
BUK2	143	87	21	12	21	2	30	4	14
JUZ	143	87	20	11	21	4	30	4	14
CHA	143	87	20	11	21	4	30	4	14
ADG1	143	87	20	11	21	4	30	4	14
ADG2	143	87	20	11	21	4	30	4	14
TBR	143	87	20	11	21	4	30	4	14
CUR	143	87	20	11	21	4	30	4	14

All the plastomes have 143 genes in total, with 87 genes in the LSC region, 20 in the IRb, 11 in the SSC, and 21 in the IRa, and four in junction sites, except BUK2. The BUK2 plastome has 21 genes in the IRb, 12 in the SSC region and only two genes in the junction sites. All the plastomes have 30 unique tRNA and four unique rRNA, and 14 intron regions.

### Presence of variants

**Fig 3:**
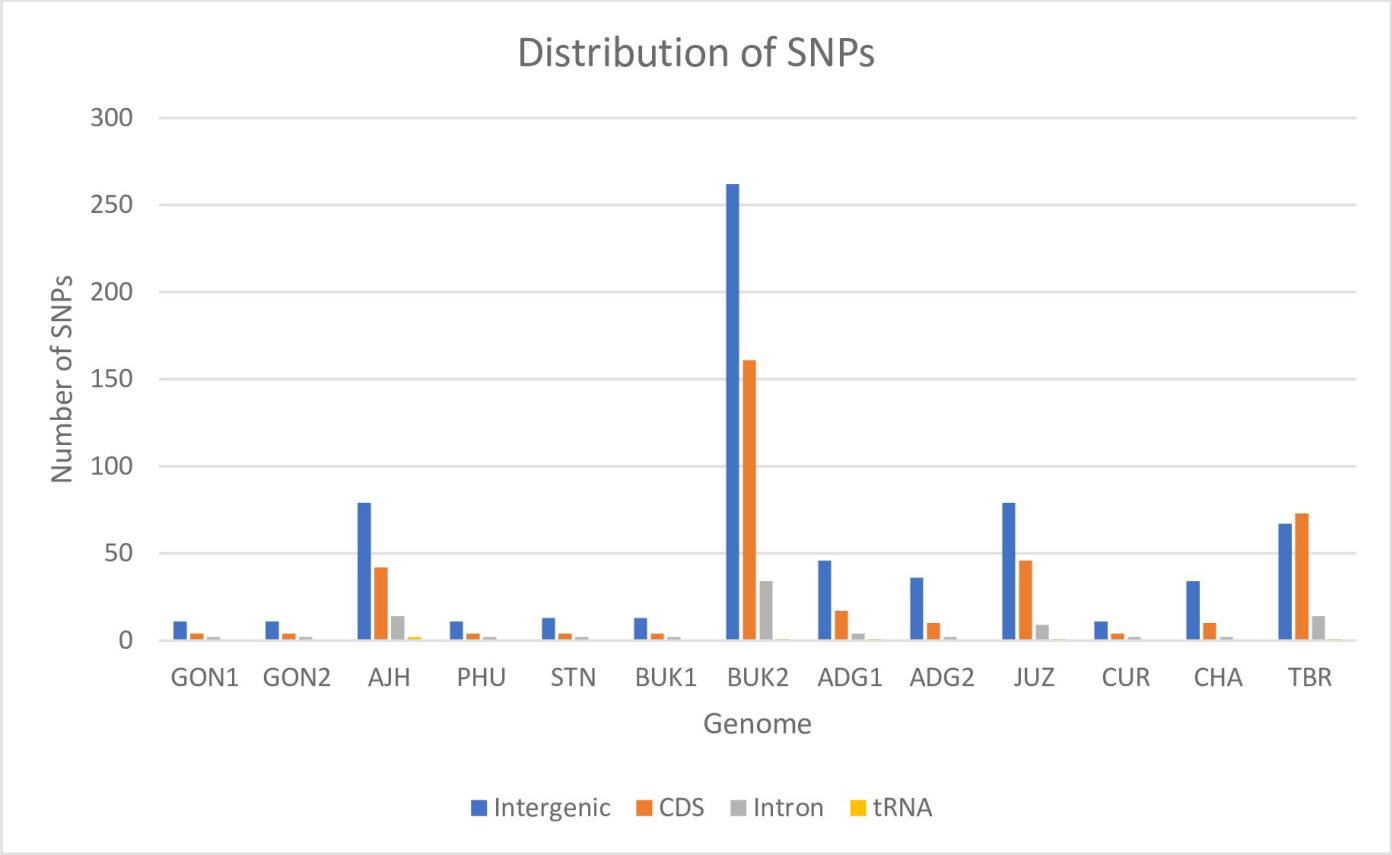
Distribution of SNPs in a panel of 13 potato plastomes. The number of SNPs and their distribution across different regions show that the majority of the SNPs are present in intergenic regions. However, the BUK2, AJH, JUZ, and TBR plastomes also have many SNPs in the CDS regions.

Despite the highly conserved nature of the plastomes, noticeable variations are present in closely related species. The multiple sequence alignment of the 13 complete chloroplast sequences reveals a great number of single nucleotide polymorphisms (SNPs) and insertions/deletions (InDels), even though they are structurally highly similar. A total of 746 SNP sites are detected and out of these, 563 are singleton variable sites (two variants), 178 are parsimony-informative sites (two variants), two are singleton variable sites (three variants), and three are parsimony-informative sites (three variants). Among the 13 plastomes, BUK2 has the highest number of SNPs compared to a majority call reference with 458 SNP sites, followed by the TBR, AJH, and JUZ, whose plastomes have 155, 137, and 135 SNPs, respectively. More than 50% of the SNPs are present in intergenic and intron regions (Fig 3). The genes harboring SNPs are listed in Table 4. Out of 746 overall SNP sites, only 57 SNPs are located in IR regions indicating that IR regions are more conserved than the LSC region with 519 SNPs and the SSC region with 170 SNPs. These results are in agreement with other studies [5, 8]. Overall, 57 genes in total have SNPs, and a few genes have more SNPs than others. The *ycf1* gene has the highest number of SNPs (55 SNPs), followed by the *ndhA* gene with 30 SNPs, *clpP* and *ndhF* genes with 18 and 16 SNPs respectively (S1 Fig).

**Table 4:** Genes harboring SNPs and InDels.

	Genes harboring SNPs, and InDels		
	SNPs	Insertions	Deletions
GON1	*atpB*, *atpF*, *ndhA*, *rps3*, *rps4*, *ycf1*	-	-
GON2	*atpB*, *atpF*, *ndhA*, *rps3*, *rps4*, *ycf1*	-	-
AJH	*atpA*, *atpB*, *atpE*, *atpF*, *ccsA*, *cemA*, *clpP*, *matK*, *ndhA*, *ndhC*, *ndhD*, *ndhF*, *ndhG*, *psaA*, *psbB*, *psbC*, *rbcL*, *rpl14*, *rpl22*, *rpoB*, *rpoC1*, *rps11*, *rps16*, *rps3*, *trnQ-UUG*, *trnR-UCU*, *ycf1*, *ycf3*	-	clpP
PHU	*atpB*, *atpF*, *ndhA*, *rps3*, *rps4*, *ycf1*	-	-
STN	*atpB*, *atpF*, *ndhA*, *rps3*, *rps4*, *ycf1*	-	-
BUK1	*atpB*, *atpF*, *ndhA*, *rps3*, *rps4*, *ycf1*	-	-
BUK2	*accD*, *atpA*, *atpB*, *atpF*, *atpH*, *atpI*, *ccsA*, *clpP*, *matK*, *ndhA*, *ndhC*, *ndhD*, *ndhF*, *ndhG*, *ndhH*, *ndhJ*, *petA*, *petB*, *petD*, *psaA*, *psaB*, *psaC*, *psbA*, *psbC*, *psbH*, *psbI*, *psbT*, *psbZ*, *rbcL*, *rpl14*, *rpl16*, *rpl20*, *rpl22*, *rpoA*, *rpoB*, *rpoC1*, *rpoC2*, *rps11*, *rps15*, *rps16*, *rps19*, *rps2*, *rps3*, *rps4*, *rps8*, *trnR-UCU*, *ycf1*, *ycf2*, *ycf3*	*atpF*, *clpP*, *ndhF*, *rps19*-fragment	*matK*, *ycf3*, *ycf1*
ADG1	*atpH*, *ccsA*, *clpP*, *ndhA*, *ndhC*, *rpoB*, *rpoC1*, *rpoC2*, *rps4*, *trnL-UAG*, *ycf1*	*ndhA*, *ycf1*	*ClpP*
ADG2	*ccsA*, *cemA*, *ndhA*, *ndhF*, *rpoB*, *rps15*, *ycf1*, *ycf2*	*-*	*clpP*
JUZ	*atpB*, *atpF*, *ccsA*, *cemA*, *clpP*, *ndhA*, *ndhC*, *ndhD*, *ndhF*, *ndhG*, *ndhH*, *petA*, *psaA*, *psbB*, *psbC*, *rbcL*, *rpl14*, *rpl22*, *rpoB*, *rpoC1*, *rps11*, *rps16*, *rps3*, *trnR-UCU*, *ycf1*, *ycf3*	*-*	*clpP*
CUR	*atpB*, *atpF*, *ndhA*, *rps3*, *rps4*, *ycf1*	*-*	*-*
CHA	*ccsA*, *cemA*, *ndhA*, *ndhF*, *rpoB*, *rps15*, *ycf1*, *ycf2*	*-*	*clpP*
TBR	*atpA*, *atpB*, *ccsA*, *clpP*, *matK*, *ndhA*, *ndhC*, *ndhD*, *ndhF*, *ndhG*, *ndhH*, *psaA*, *psbA*, *psbC*, *psbI*, *rbcL*, *rpl14*, *rpl20*, *rpl22*, *rpoB*, *rpoC1*, *rpoC2*, *rps11*, *rps15*, *rps3*, *rps8*, *rrn23*, *trnR-UCU*, *ycf1*, *ycf1-fragment*, *ycf2*, *ycf3*	*clpP*	*clpP*

The SNPs and InDels were annotated. The BUK2, TBR, AJH, and JUZ plastomes have higher number of genes with SNPs compared with the other plastomes. The GON1, GON2, PHU, ST, BUK, and CUR plastomes lack InDels. Comparatively, the SNPs are more common than the InDels in all plastomes.

**Fig 4:**
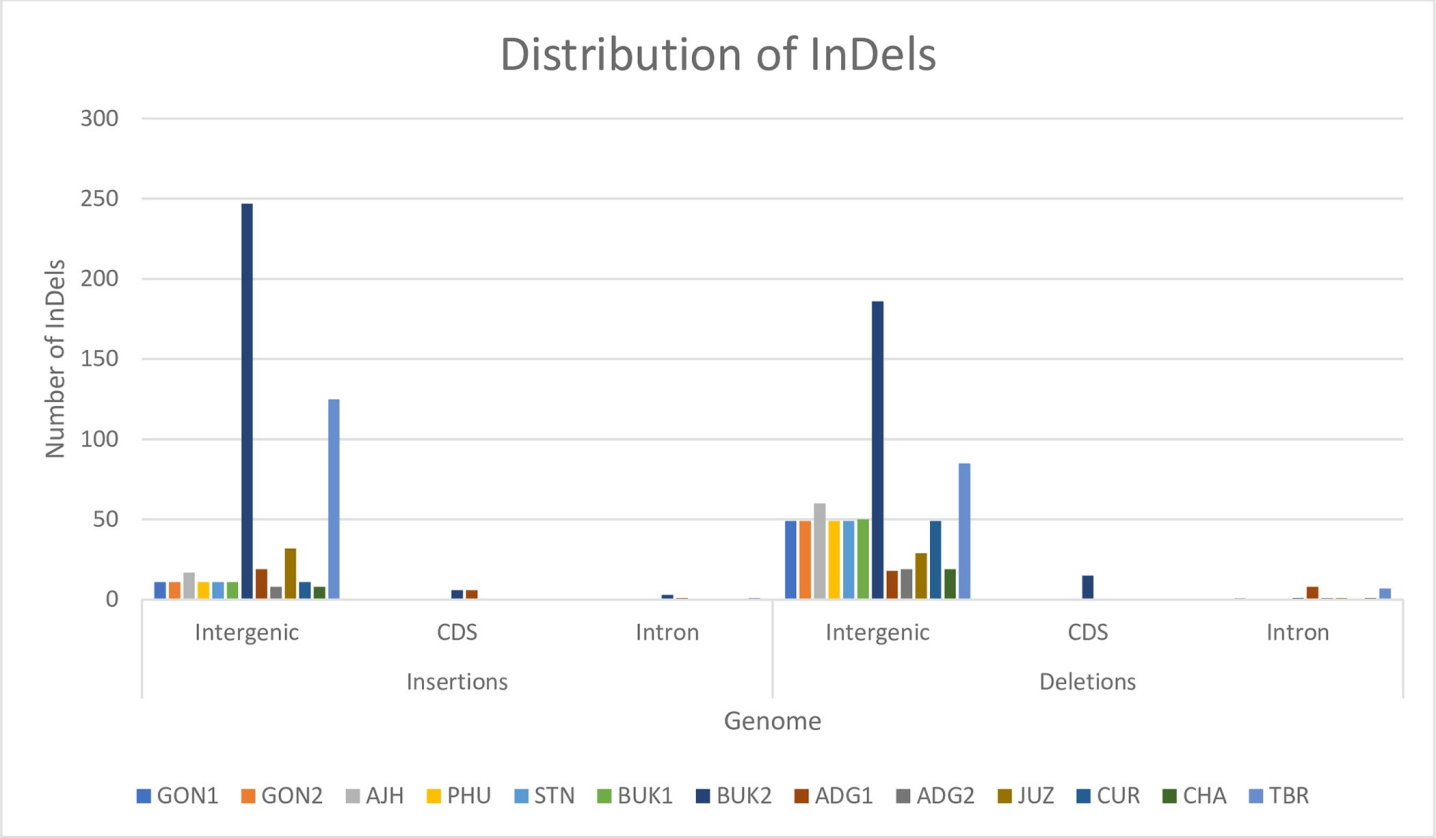
Distribution of InDels in a panel of 13 potato plastomes. Comparatively, BUK2 and TBR have the greatest number of InDels of all the plastomes. More than 90% of the InDels are present in intergenic regions.

Similarly, InDels were detected from the multiple sequence alignment of the plastomes. Overall, 790 InDels are present of which 413 are insertions and 377 are deletions. There are more deletions than insertions in the plastomes of GON1, GON2, AJH, PHU, STN, BUK1, ADG2, CHA, AND CUR, while those of TBR, JUZ, and BUK2 contain more insertions than deletions. Overall, BUK2 and TBR have more InDels than the others with 458 and 218 InDels, respectively. Annotation shows that more than 90% of these InDels are present in intergenic and intron regions, which may have significant impact on gene expression regulation. Only a small number of InDels are present in genes (Fig 4 and Table 4). From a total of 790 InDels, only four InDels are present in IR regions, 714 in the LSC region, and 72 InDels in the SSC region. This further confirms the highly conserved nature of IR regions. In total, only four genes have deletions and six genes have insertions. The *clpP* gene has the highest number of InDels with 17 InDels in its intron region. Also, the *ycf1* and the *matK* genes have 12 and nine InDels, respectively (S2 Fig). High divergence of the *ycf1* gene has been observed in the plastomes of many plant species and this has led to it being used as a DNA barcode in land plants [5, 32].

A few length mutations are also present, some of them conserved in multiple plastomes. A 48 bp deletion is present in the GON1, GON2, PHU, STN, BUK1, and CUR plastomes and this is the largest deletion found in the panel. It is present in the intergenic region between *rps16* and *trnQ-UUG* genes in the LSC region. Similarly, a 10 bp deletion is present in AJH, ADG1, ADG2, JUZ, CHA, and TBR in the intergenic region between *ndhG* and *ndhI* genes in the SSC region. Likewise, the second largest deletion of 30 bp is present in the AJH plastome in the intergenic region between the *trnS-GCU* and *trnG-UCC* genes in the LSC region. BUK2 also has 30 and 18 bp deletions in the intergenic region of LSC, and a 9 bp and 6 bp deletion in the *matK* and *ycf1* genes, respectively.

Moreover, a 7 bp insertion is located in the intergenic region between *rbcL* and *accD* genes in GON1, GON2, PHU, STN, BUK1, and CUR. Similarly, AJH, JUZ, TBR, and BUK2 have a 7 bp insertion in the intergenic region between *psaA* and *ycf3* genes. The largest insertion of 55 bp is in the BUK2 plastome in the intergenic region between the *rpl20* and *rps12* genes. The TBR plastome has 30 bp and 27 bp inserted in the intergenic region between *trnG-UCC*, *trnR-UCU* and *rps16*, *trnQ-UUG* genes, respectively. Many of these length mutations are present in the intergenic regions, hence they have not altered the gene content nor the overall structure of the 13 plastomes.

### Repeat analyses

**Fig 5:**
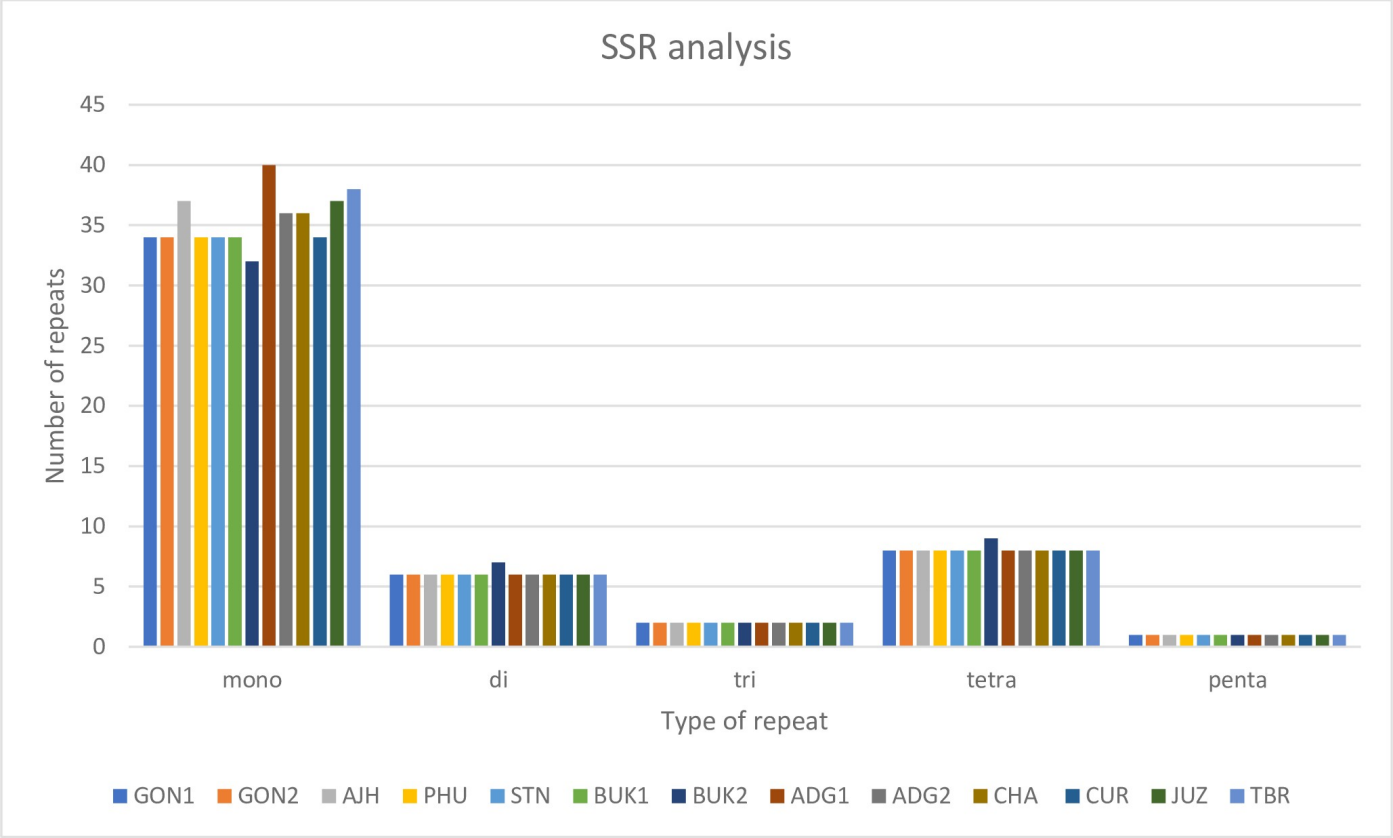
Analysis of SSRs in a panel of 13 potato plastomes. Five SSRs were identified in all the plastomes, namely mononucleotide, dinucleotide, trinucleotide, tetranucleotide, and pentanucleotide repeats. Mononucleotide repeats are the most abundant.

Simple sequence repeats (SSRs) are important molecular genetic markers that are extensively used in populations genetics, evolutionary studies, diversity assessments, and ecological studies [13]. Between 51 to 57 SSRs are present in each of the 13 plastomes, ranging from mononucleotide repeats to pentanucleotide repeats. The mononucleotide repeat abundance is the highest amongst the SSRs for all the plastomes (Fig 5). The GON1, GON2, PHU, STN, BUK1, BUK2, and CUR plastomes each have 51 SSRs in total, of which 34 (67%) are mono-repeats. While all of them have the same number of di-, tri-, tetra-, and pentanucleotide repeats (except for BUK2, which has one extra di- and tetra- repeat), they differ in mono-repeats. BUK2 and ADG1 have the lowest and highest number of mono-repeats containing 32 and 40 repeats, respectively. Similarly, all of the dinucleotide repeats are composed of AT/TA repeats, and 50% of trinucleotide repeats are TTA, indicating an abundance of AT bases in chloroplast SSR sequences.

**Fig 6:**
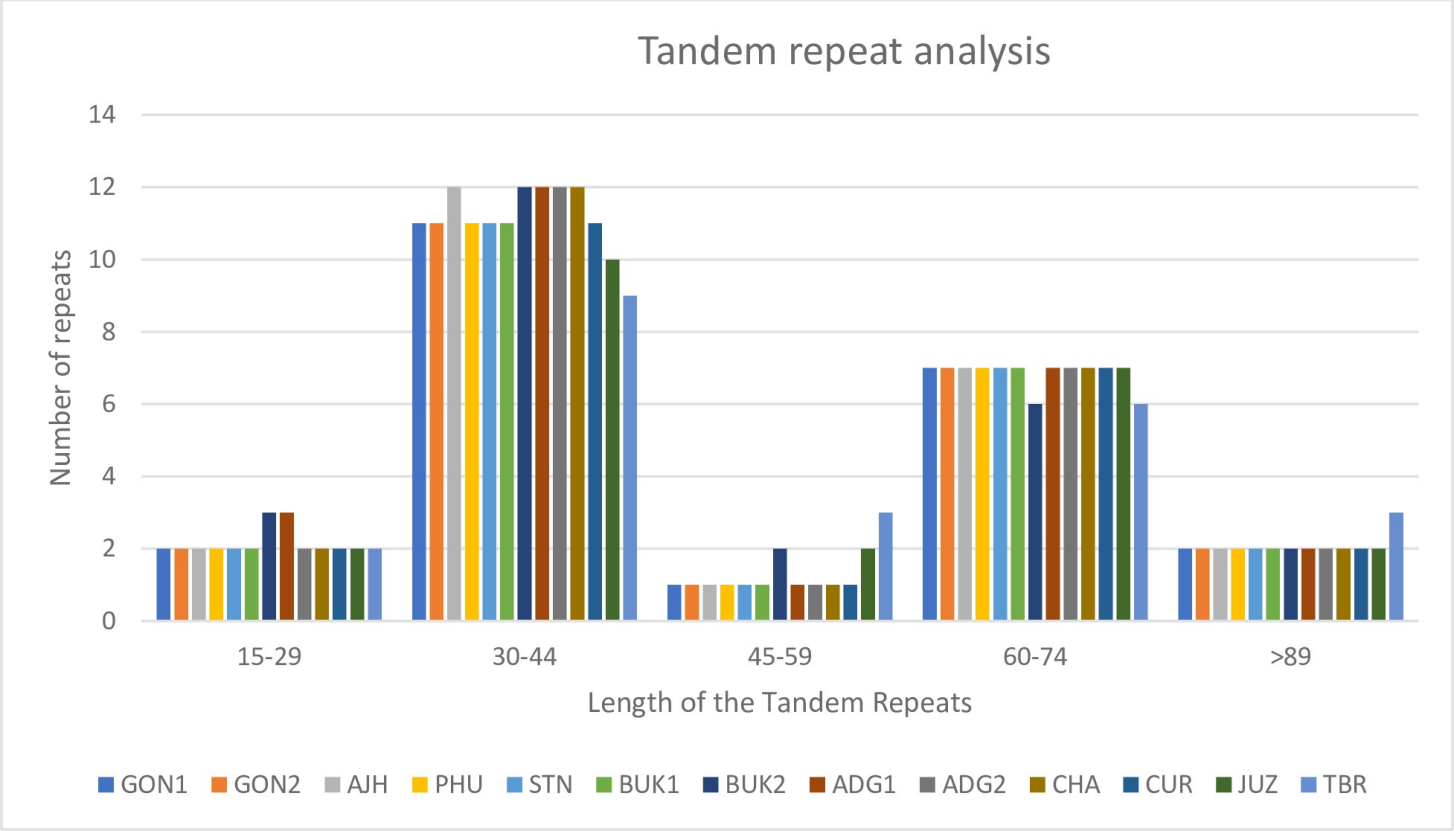
Analysis of tandem repeats in a panel of 13 potato plastomes. Overall, 23 to 25 tandem repeats are present in all the plastomes and their sizes range from 26 bp to 91 bp. The size of the repeats is divided into five different ranges and number of repeats present in that particular range are presented here. Most of the repeats have a size in the range of 30–44 bp.

Also, 23–25 tandem repeats are present in all of them and the lengths of tandem repeats range from 26 bp to 91 bp. Overall, GON1, GON2, PHU, STN, BUK1, JUZ, TBR, and CUR have 23 tandem repeats, AJH, CHA, and ADG2 have 24 tandem repeats and ADG1, BUK2 have 25 repeats. The length of these repeat sequences is mostly in the range of 30–44 bp. Three tandem repeat sequences in TBR are 90 bp large and the other 12 plastomes have two 90 bp tandem repeat sequences (Fig 6). Similarly, 31–35 repeat sequences are present in all of them, including forward, palindromic, reverse, and complementary repeat sequences. Overall, 31 direct and palindromic repeat sequences are present in GON1, GON2, AJH, PHU, STN, BUK1, ADG1, ADG2, CHA, and CUR. Similarly, 32, 33, and 35 direct and palindromic repeat sequences are present in JUZ, TBR and BUK2 respectively. However, BUK2 is the only one that has two reverse and one complementary repeat sequences. All plastomes have 16 palindromic repeats, and they range from 56 bp to 30 bp, while the forward repeats range from 61 bp to 30 bp.

### Identification of chloroplast DNA types

Potato has a diverse cytoplasm with different plastome types, which have been previously demonstrated to affect agronomic traits [33]. Some of the cytoplasm types in potato have been shown to induce higher percentages of tuberization, higher tuber yields, and earlier vine maturity [33]. In order to study the plastome diversity among the different accessions in the genome panel, their types were identified. Plastome specific markers and an *in silico* restriction enzyme analysis were used to identify plastome types for potato and the results were compared with previous studies [12, 29]. The plastome types of the GON1, GON2, PHU, STN, BUK1, CUR, ADG1, AJH, and JUZ accessions were positively identified, whereas the rest of the plastomes could not be clearly identified with the marker analysis due to the lack of supporting data from previous studies. The GON1, GON2, PHU, STN, BUK1, and CUR plastomes are of S-type, which is the most commonly found in *Solanum stenotomum* species, whereas ADG1, AJH, and JUZ are found to be of the C-type (Table 5). Both S and C-types are rarely found in modern potato cultivars, and instead are predominantly found in landraces or wild species [33]. Our results confirm these earlier findings.

**Table 5:** Identifying plastome types from marker analysis.

Genome	NTCP6	NTCP7	NTCP8	NTCP9	NTCP12	NTCP14	NTCP18	cp_type
GON1	127	173	251	289	129	150	186	**S**
GON2	127	173	251	289	129	150	186	**S**
PHU	127	173	251	289	129	150	186	**S**
STN	127	173	251	289	129	150	186	**S**
BUK1	127	173	251	289	129	150	186	**S**
CUR	127	173	251	289	129	150	186	**S**
ADG1	173	174	251	289	126	150	187	**C**
ADG2	174	174	250	289	127	151	186	**C/A**
CHA	174	174	250	289	127	151	186	**C/A**
AJH	174	174	249	289	127	151	186	**C**
JUZ	174	174	250	289	126	150	186	**C**
TBR	174	174	255	310	125	150	188	**inconclusive**
BUK2	174	174	253	249	128	154	186	**inconclusive**

The *in silico* PCR product size for each marker used are presented here. The respective plastome type was determined after comparison with previous studies. The ADG2 and CHA plastomes are identified as ambiguous between C/A type cpDNAs due to lack of supporting data from the previous marker analysis studies, while TBR and BUK2 are inconclusive.

The chloroplast DNA types of ADG2, CHA, TBR, and BUK2 were instead identified by *in silico* restriction endonuclease analysis. The restriction fragment patterns found were compared with the previous studies to identify plastome types [10]. GON1, GON2, PHU, STN, BUK1, CUR, ADG1, AJH, JUZ, CHA, and ADG2 plastomes gained a 3769 bp fragment when digested with *BamHI*, which indicates that these plastomes are either S-type, C-type, or the A-type. Loss of a 48 bp fragment, when digested with *HindIII*, confirms GON1, GON2, PHU, STN, BUK1, and CUR as the S-type. Similarly, loss of a ~300 bp fragment when digested with *BamHI* confirms ADG2 and CHA as A-type. No loss of any fragment when digested with *BamHI* and *HindIII* confirms ADG1, AJH, and JUZ as C-type plastome. Since none of these changes were observed in TBR, it is a W-type. Similarly, since BUK2 also does not have any of these changes except for a gain of a 20.9 kb fragment when digested with *PvuII*, suggests BUK2 has a W2-type chloroplast DNA (Table 6).

**Table 6:** Restriction endonuclease analysis and polymorphisms in fragment length.

Genome	*Bam*HI	*Hind*III	*Kpn*I	*Pvu*II	*Xho*I	cp type
**GON1, GON2, PHU, STN, BUK, CUR**	15.6 kb + 3.76 kb → 19.4 kb	2.58 kb—48 bp → 2.54 kb	-	-	-	**S**
**ADG2, CHA**	15.6 kb + 3.76 kb → 19.4 kb & 3.88 kb—297 bp → 3.59 kb	-	-	-	-	**A**
**ADG1, AJH, JUZ**	15.6 kb + 3.76 kb → 19.4 kb	-	-	-	-	**C**
**TBR**	-	-	-	-	-	**W**
**BUK2**	-	-	-	2 X 20.9 kb → 41.8 kb	-	**W2**

Different polymorphisms were observed in fragment lengths when digested with five restriction enzymes. These length polymorphisms were compared with previous studies to identify plastome types [1, 10]. The RFLP analysis confirms the findings from the marker analysis and also identify the types of ADG2, CHA, BUK2, and TBR.

The cultivated diploid species GON1, GON2, PHU, and STN all have the S-type plastome. The same classification was observed for a majority of these accessions in prior studies [1, 10, 12]. However, we show that BUK1 and BUK2, the two plastomes from the wild diploid species (same accession), have S-type and W2-type cpDNAs, respectively. The majority of *Solanum bukasovii* accessions studied previously were shown to have the C-type, though four accessions of *S*. *bukasovii* were previously demonstrated to have the S-type [11, 34]. A W- type was shown in two accessions of *S*. *bukasovii* [11, 34], but a W2-type of *S*. *bukasovii* cpDNA has not been mentioned in previous studies. Also, the W2-type has been seen in other wild accessions from potato species [1]. This diversity of plastome types in *Solanum bukasovii* is likely due to the successful domestication and parallel differentiation from time to time and place to place from ancestral species [11].

*S*. *curtilobum*, CUR, which is a cultivated pentaploid, has an S-type plastome like the cultivated diploids. This is in agreement with previous studies [10, 12]. CHA and ADG2 have an A-type plastome, while ADG1 has the C-type. The differences in types of the two *S*. *tuberosum* subsp. *andigena* accessions (ADG1 and ADG2) were reported, having either A, C, or an S-type [1]. Similarly, the A-type was found previously in a few accessions of *Solanum chaucha* (CHA) [12]. The cultivated diploid AJH and the triploid JUZ also have the C-type plastome. These results are in agreement with the previous studies where *S*. *xajanhuiri* (AJH) and *S*. *juzepczukii* (JUZ) species were shown to have the C-type [12, 34]. Finally, the tetraploid TBR has a W-type cpDNA, which was previously only observed in one variety of *S*. *tuberosum* subsp. *tuberosum* [10]. The T-type plastome is more commonly found in the cultivated Chilean potato *S*. *tuberosum* subsp. *tuberosum*. It is interesting that the accession of *S*. *tuberosum* subsp. *tuberosum* in our panel has a W-type plastome, and it may partially explain its close relatedness with wild potato species [14].

### Polymorphisms responsible for the divergence of chloroplast DNA types

According to the initial study that presented the method of classifying plastome types by restriction endonuclease analysis [10], the W-type is the most primitive type and other plastome types are derived from this. It was shown that other plastome types were derived by a single change in the restriction fragment pattern, except for the T-type. Here, we discuss in detail the point mutations that are responsible for the type divergence. It was shown *in vitro* that the W2-type (sometimes shown as W^”^) was derived from the W-type with a single change in the *PvuII* restriction fragment pattern that led to a 21.2 kb fragment [1, 10]. Here, we show that it is the single point mutation in the *PvuII* restriction site present in the *rps11* gene in the LSC region that is responsible for the origin of the W2-type. The BUK2 plastome has a change of **A** to **G** in the *PvuII* restriction site, which made BUK2 gain a fragment of 20.9 kb (S3 and S4 Figs). Likewise, gain of a 3.66 kb fragment was observed in A, S, and C-type plastomes when *in vitro* digested with *BamHI* [10]. Here, we show that a single polymorphism in the *BamHI* restriction site at the intergenic region between *cemA* and *petA* genes in the LSC region is responsible for this gain (S3 Fig). A gain of 3.76 kb fragment was observed in GON1, GON2, AJH, PHU, STN, BUK, CUR, ADG1, ADG2, CHA, and JUZ plastomes with a change of nucleotide from **G** to **A**, indicating either A, S, or C-type (S4 Fig).

**Fig 7:**
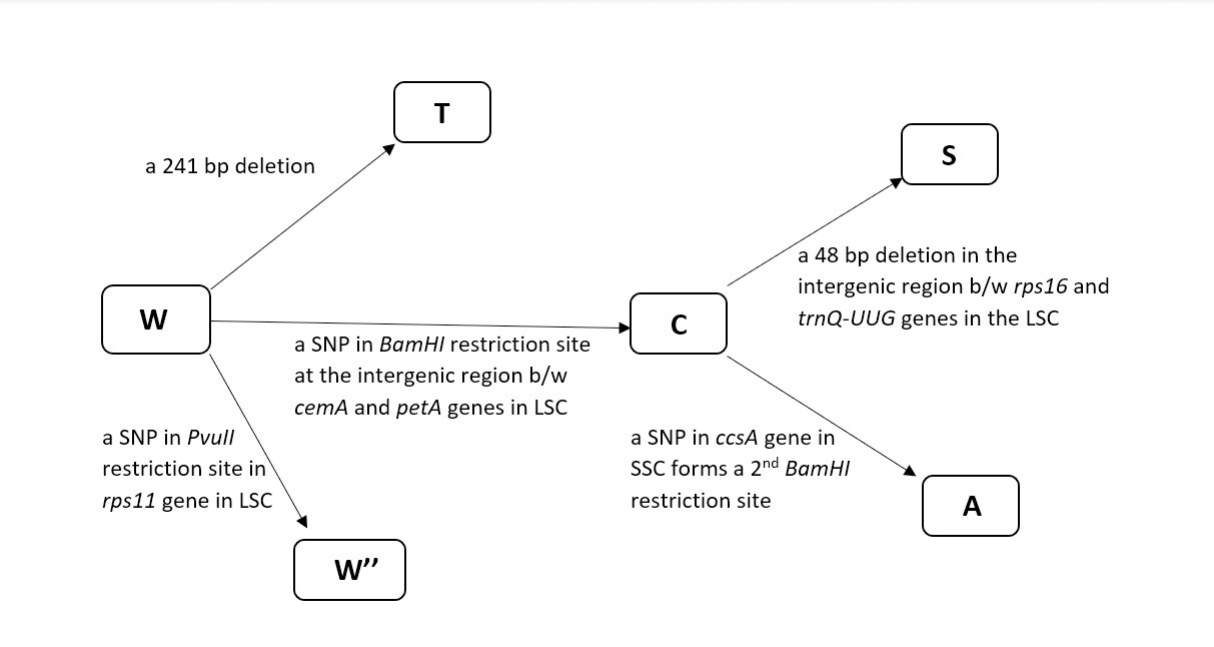
Schematic representation of evolution of cpDNA types in potato. The W-type is the primitive type of potato plastome from which other types were derived. A 241 bp deletion in the W-type gave rise to the T-type cpDNA [11]. The W2 (W”) is derived from W due to a single nucleotide change. Similarly, the C-type is derived from the W-type by a single nucleotide change which further formed the S- and A-type plastomes. The A-type derived from the C-type due to the formation of a second restriction site by an SNP, while a 48 bp deletion in the C-type led to the S-type.

Furthermore, a single site change **(G** to **C)** in the coding region of the *ccsA* gene in the SSC region forms a second *BamHI* restriction pattern (ggatcc), as observed in ADG2 and CHA (S3 Fig). This led to these plastomes losing a ~300 bp fragment (S4 Fig). Similarly, a loss of ~350bp fragment was used to distinguish type-A from type-C when digested with *BamHI* [10]. Finally, a 48 bp deletion mentioned above in GON1, GON2, PHU, STN, BUK1, and CUR in the LSC region makes the *HindIII* digested fragment 48 bp shorter (S3 and S4 Figs). This loss of 48 bp distinguishes the S-type from the C-type, as mentioned in the previous studies [10]. Also, the PCR product size of the NTCP6 marker can distinguish the S-type from C-type—a product of 127 bp was observed for the S-type plastomes, corresponding to products of 173 and 174 bp in other types. Hence, it clearly shows that a deletion of ~48 bp in the above-mentioned region confirms a plastome to be an S-type. No T-type plastome was found in these 13 potato accessions, but a 241 bp deletion is commonly used to identify this type [11]. These particular polymorphic regions will greatly aid in plastome type identification (Fig 7).

### Phylogenetic analysis

**Fig 8:**
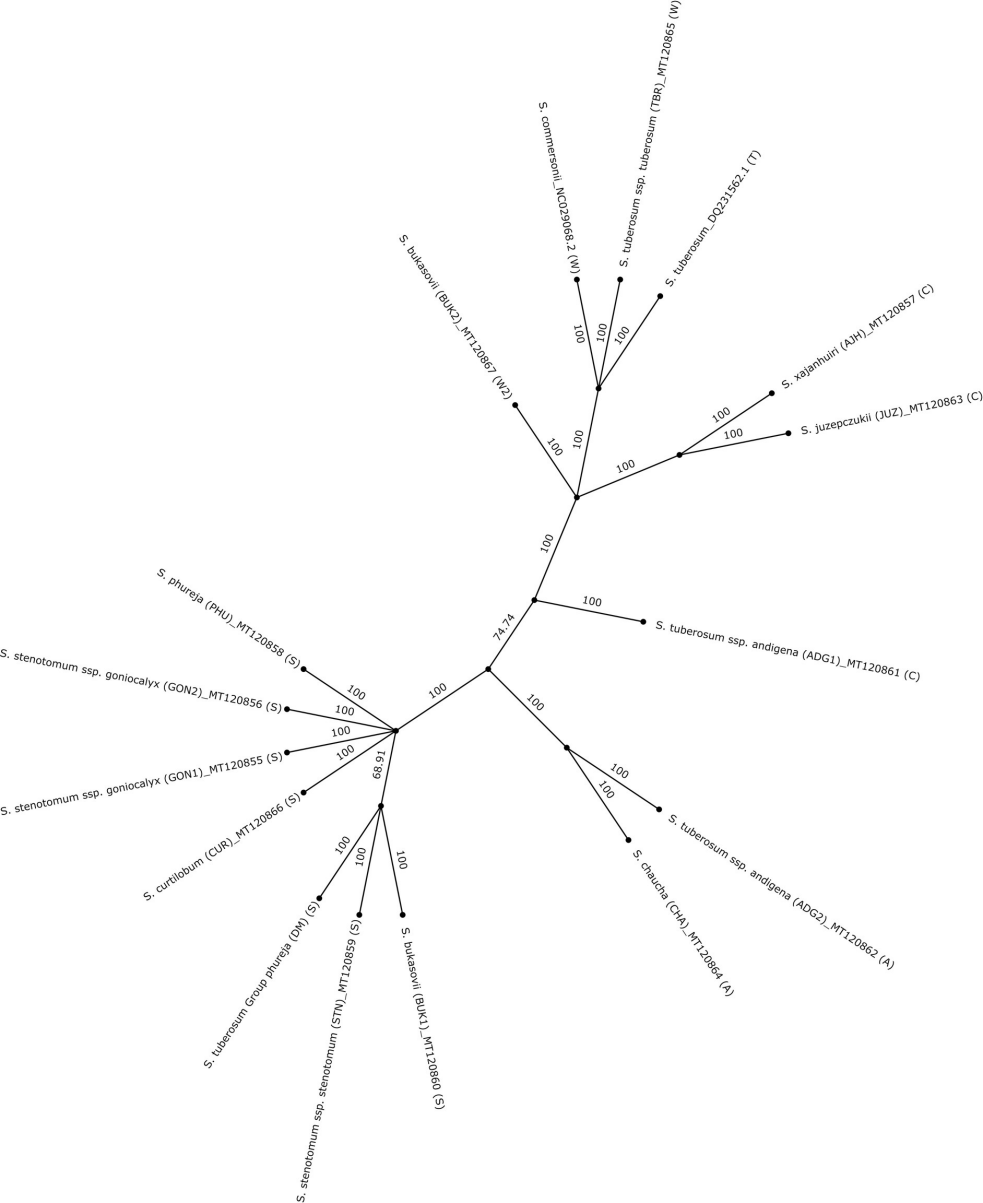
Phylogenetic tree of 16 plastomes. A phylogenetic tree was constructed using complete plastomes of sixteen potato accessions (without the IRb repeat). Plastome types of three other potato species were also determined (types are shown in brackets at the end). It is interesting to see that all the plastomes are grouped together according to their types.

Twelve of the potato accessions in the panel (BUK2 is the exception), were previously classified by their nuclear genomes [14]. Here, the complete sequences of their plastomes (with the addition of BUK2) were analyzed to understand their phylogenetic relationships relative to the nuclear genome classification. The IRb region was omitted to reduce redundancy and bias. In addition, the plastomes of DM, *S*. *commersonii*, and *S*. *tuberosum* [3, 4, 18], all of which are publicly available, were also included in the phylogeny (Fig 8). All the plastomes group together according to their type. Most of the branches and nodes in the phylogeny are strongly supported by the bootstrap analysis, while the nodes at DM, BUK1, and STN, and the node separating S-type plastome with other types have moderate support. All the cultivated diploids with an S-type plastome are grouped into a single clade, where also BUK1 groups, despite being a wild species. It is interesting to see that the plastome of DM, a double monoploid derived from *S*. *tuberosum* group *Phureja* clone, is in the same clade as that of PHU [18] (Fig 8). The plastome of a cultivated pentaploid species, CUR, is grouped together with the diploid species. Similar results were observed previously but the reason for this is not known, however it can be presumed that *S*. *curtilobum* could be derived from a cross between *S*. *juzepcuzkii* as a male parent and a cultivated diploid species as a female parent [10]. The plastomes of ADG1 and ADG2 are present in different clades and this is clearly due to the difference in their type. However, the plastome of CHA, a triploid species, is closely related to that of ADG2. This is reasonable since *Solanum chaucha* (CHA) is believed to be derived from an *andigena* female and a *stenotomum* male [10]. The plastomes of another cultivated diploid, AJH and the triploid JUZ are placed close to each other. *S*. *xajanhuiri* (AJH) is a bitter potato derived from *S*. *megistacrolobum*, which also has a C-type plastome [12]. Finally, BUK2, TBR, *S*. *tuberosum*, and *S*. *commersonii* plastomes are clustered together in a single clade. This clade contains the W, W2, and T-types, and previous studies have shown that they (W, and T type) group in the same clade [12]. BUK2, a diploid wild species, is close to *S*. *commersonii*, which is also a wild diploid species. Not surprising was the fact that the plastome of *S*. *commersonii* was found to be phylogenetically distinct from the other cultivated potatoes as this was previously reported in a study of analyses of plastome restriction sites [35]. Consistent with these analyses, *S*. *commersonii* and *S*. *tuberosum* (which are clustered together) are sexually compatible [36].

#### Comparison between the plastome phylogeny and nuclear genome CNV-based classification

A copy number variation (CNV) based classification of the nuclear genomes from this panel of species (except BUK2) was previously presented [14]. That study shows that GON1, GON2, PHU, STN, and ADG1 cluster together with ADG2, with CHA being close to this cluster. A similar relationship is observed in our plastome phylogeny, where ADG2, and CHA cluster closer to the cultivated diploids, GON1, GON2, PHU, and STN (Fig 8). The bitter potatoes JUZ, CUR, and AJH clustered together in the CNV based classification. However, here only JUZ and AJH were in a single cluster, but CUR is closely related to the cultivated diploids. Moreover, TBR and M6 were clustered together and BUK1 remained an outlier as observed in the CNV classification. BUK1 here is grouped together with the other diploids, whereas TBR clustered with other wild species including BUK2. Despite that plastome classification does not necessarily follow species boundaries in potato (many potato species can readily cross) [9], a strong similarity was observed between the CNV based classification [14] and the plastome classification of the selected accessions in this study. Except for the CUR and BUK1, the rest of the phylogeny is in congruence with the CNV based classification of these accessions.

## Conclusions

A panel of 13 potato plastomes were assembled and annotated. All of them are highly similar in gene content and genomic structures. This was also previously found for 202 plastomes of *Solanum* accessions in the section *Petota* [8], indicating a high degree of conservation. Nonetheless, variation is present in all the plastomes, with BUK2 having the highest number of SNPs and InDels among them. Overall, the *ycf1* gene has a greater number of variations compared to the other genes. In addition, identified SSRs, along with SNPs and InDels, can be used as genetic markers in phylogenetic analyses. Most importantly, the plastome types of all accessions were identified. Though BUK1 and BUK2 are from the same accession of the wild species *S*. *bukasovii*, they have different plastome types, which is likely due to the accession being collected as a natural population. This also illustrates *S*. *bukasovii* being a natural outcrossing wild species, and the importance of plastome analyses for better understanding of potato genetic resources. Moreover, the phylogeny of 16 complete plastome sequences reveal clustering according to the specific types. Even though some congruency has been observed between plastome and nuclear genome clustering of the accessions in the panel, organelle phylogeny does not entirely correspond to the nuclear phylogeny, which is also known from literature. Finally, a detailed description of the polymorphisms responsible for the divergence of plastome types is beneficial for quickly identifying the potato plastome types. A 48 bp deletion was found in the GON1, GON2, PHU, STN, BUK1, and CUR plastomes that is associated only with the S-type plastome. The findings from this panel of potato accessions will be helpful in future research to effectively identify plastome types in potato species, as source for information in breeding programs, as well as to further the understanding of the evolutionary history of the potato.

## Supporting information

S1 Fig Number of SNPs present in each gene from the panel of 13 potato plastomes. (PDF)

S2 Fig Number of InDels present in each gene from the panel of 13 potato plastomes. (PDF)

S3 Fig Point mutations responsible for different types of the 13 plastomes. (A) A point mutation (A to G) in the *PvuII* restriction site of the BUK2 plastome. (B) Mutation from G to A in the *BamHI* restriction site in the GON1, GON2, PHU, STN, BUK1, ADG1, ADG2, AJH, CUR, JUZ, and CHA plastomes. (C) A single nucleotide change from G to C in ADG2, and CHA genomes forms a *BamHI* restriction site. (D) A 48 bp deletion in the GON1, GON2, PHU, STN, CUR, and BUK1 plastomes.
(PDF)

S4 Fig Restriction fragment patterns in different plastome types. The restriction fragment patterns of five different plastome types found in the 13 accessions are shown here, one accession per plastome type is represented here. (A) *PvuII* restriction fragment patterns, + indicates a gain of 20.9 kb in the BUK2 plastome. (B) *BamHI* restriction fragment patterns; × indicates a gain of 3.76 kb in all the plastomes except TBR, and BUK2. ≠ indicates a loss of ~300 bps from a 3.88 kb fragment. (C) *HindIII* restriction patterns;♦indicates a loss of 48 bps in the 2.58 kb fragment. The circles indicate loss of fragments in BUK2 plastome which were never reported.
(PDF)
