# Supplementary figures and images for: Complete plastome assemblies from a panel of 13 diverse potato taxa
Source: PLoS One. 2020 Oct 8;15(10):e0240124. doi: 10.1371/journal.pone.0240124 (PMC7544113; doi:10.1371/journal.pone.0240124)

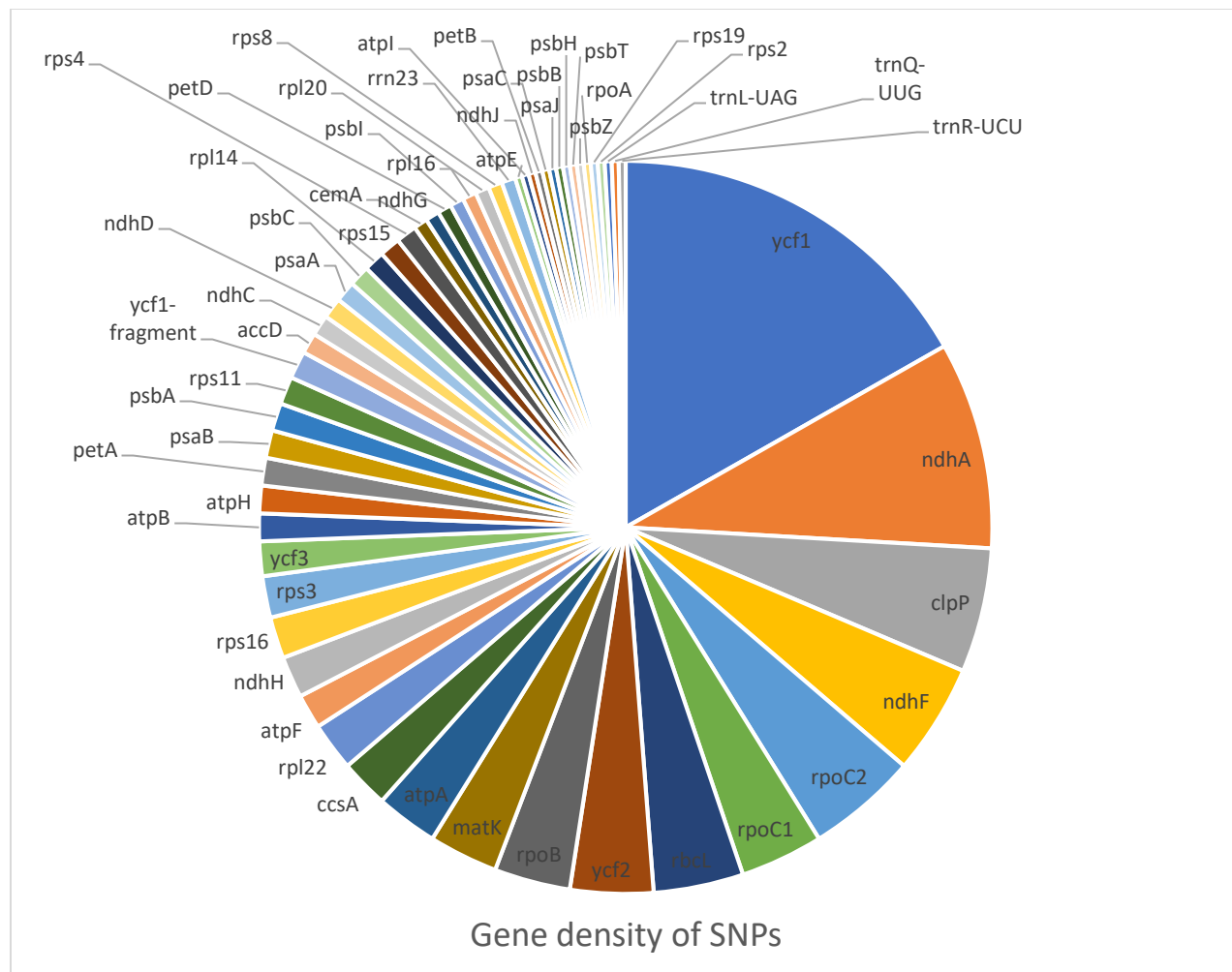

Supplement: S1 Fig — (PDF) [file pone.0240124.s001.pdf]

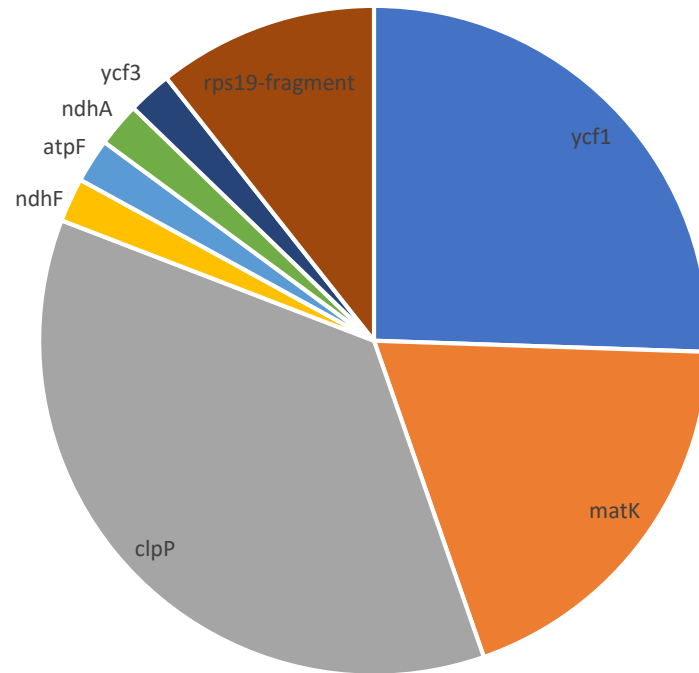

Gene density of InDels

Supplement: S2 Fig — (PDF) [file pone.0240124.s002.pdf]
