# Supplementary material for: Complete plastome assemblies from a panel of 13 diverse potato taxa
Source: PLoS One. 2020 Oct 8;15(10):e0240124. doi: 10.1371/journal.pone.0240124 (PMC7544113; doi:10.1371/journal.pone.0240124)
Supplement: S3 Fig — (A) A point mutation (A to G) in the PvuII restriction site of the BUK2 plastome. (B) Mutation from G to A in the BamHI restriction site in the GON1, GON2, PHU, STN, BUK1, ADG1, ADG2, AJH, CUR, JUZ, and CHA plastomes. (C) A single nucleotide change from G to C in ADG2, and CHA genomes forms a BamHI restriction site. (D) A 48 bp deletion in the GON1, GON2, PHU, STN, CUR, and BUK1 plastomes. (PDF) [file pone.0240124.s003.pdf]

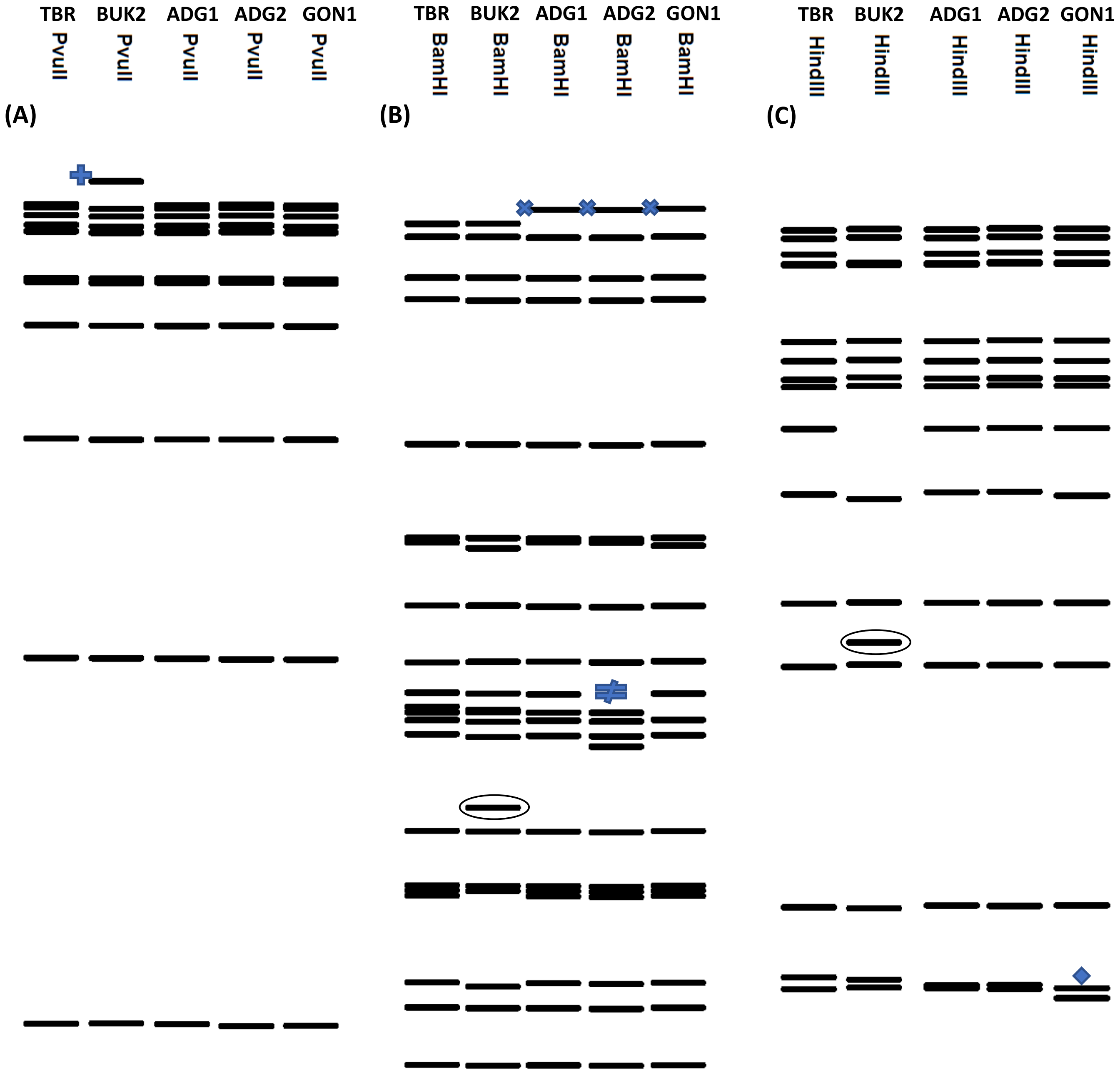
TBR BUK2 ADG1 ADG2 GON1
PvuII PvuII PvuII PvuII PvuII
(A)
TBR BUK2 ADG1 ADG2 GON1
BamHI BamHI BamHI BamHI BamHI
(B)
TBR BUK2 ADG1 ADG2 GON1
HindIII HindIII HindIII HindIII HindIII
(C)
