# Supplementary material for: Complete plastome assemblies from a panel of 13 diverse potato taxa
Source: PLoS One. 2020 Oct 8;15(10):e0240124. doi: 10.1371/journal.pone.0240124 (PMC7544113; doi:10.1371/journal.pone.0240124)
Supplement: S4 Fig — The restriction fragment patterns of five different plastome types found in the 13 accessions are shown here, one accession per plastome type is represented here. (A) PvuII restriction fragment patterns, + indicates a gain of 20.9 kb in the BUK2 plastome. (B) BamHI restriction fragment patterns; × indicates a gain of 3.76 kb in all the plastomes except TBR, and BUK2. ≠ indicates a loss of ~300 bps from a 3.88 kb fragment. (C) HindIII restriction patterns;♦indicates a loss of 48 bps in the 2.58 kb fragment. The circles indicate loss of fragments in BUK2 plastome which were never reported. (PDF) [file pone.0240124.s004.pdf]

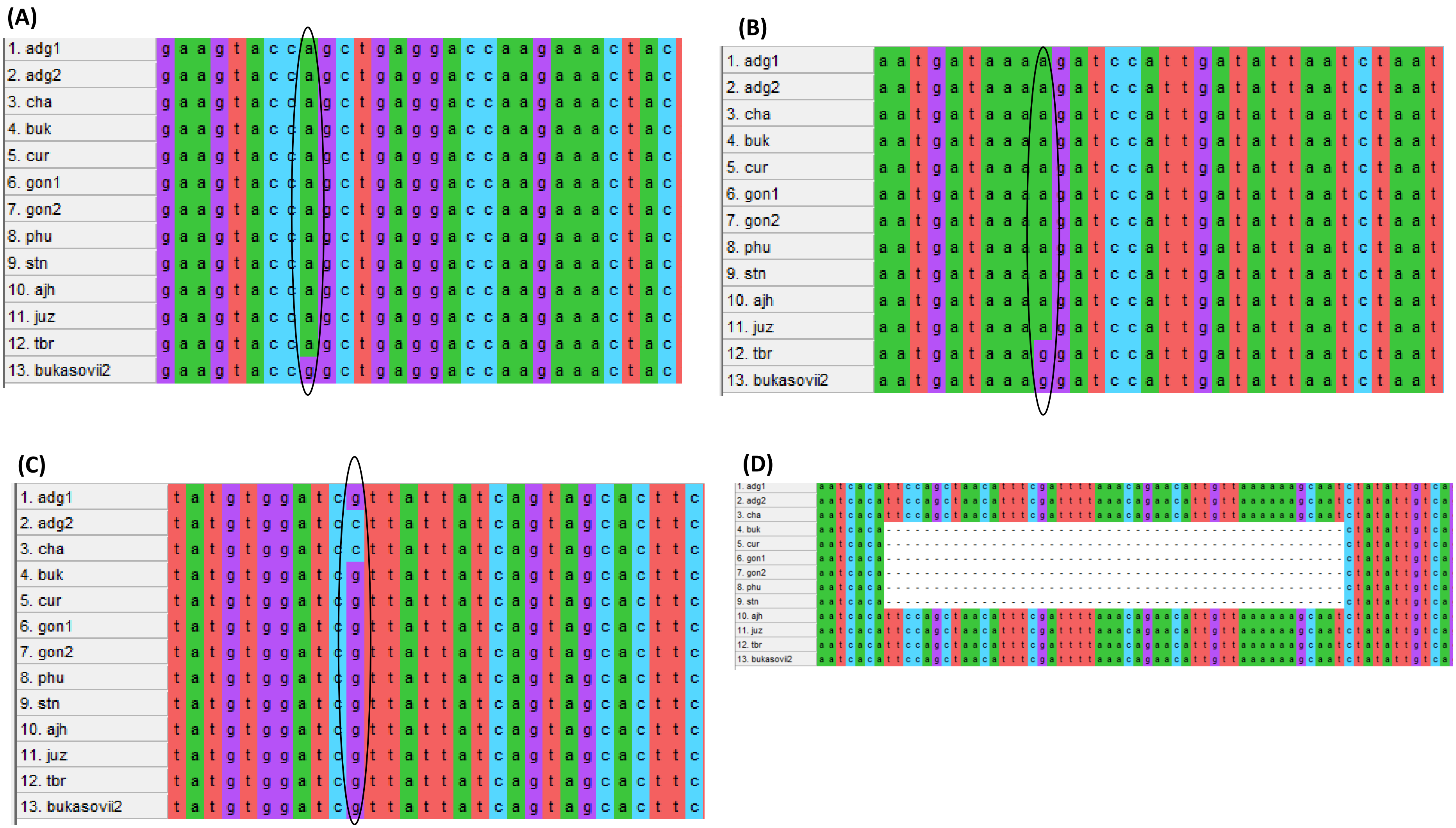
(A)
(B)
(C)
(D)
1. adg1
2. adg2
3. cha
4. buk
5. cur
6. gon1
7. gon2
8. phu
9. stn
10. ajh
11. juz
12. tbr
13. bukasovii2
